# Evaluation of the feasibility and effectiveness of trauma-focused cognitive behavioural therapy for children and youth in Ukraine during the war

**DOI:** 10.1192/j.eurpsy.2025.10032

**Published:** 2025-07-01

**Authors:** Elisa Pfeiffer, Maike Garbade, Renée Beer, Anette Birgersson, Natalie Cabrera, Judith A. Cohen, Esther Deblinger, Rafaela Gjini, Veronica Kirsch, Zlatina Kostova, Michael Larsson, Anthony Mannarino, Gavin Moffitt, Marja Onsjö, Tale Ostensjo, Anna Vikgren, Hanna Weyler, Vitalii Klymchuk, Cedric Sachser

**Affiliations:** 1 Clinic for Child and Adolescent Psychiatry, Psychosomatics and Psychotherapy, Ulm University, Ulm, Germany; 2 German Center for Mental Health (DZPG), Partner Cite Ulm, Ulm, Germany; 3 Department of Clinical Psychology and Child and Adolescent Psychotherapy, Catholic University Eichstätt-Ingolstadt, Ingolstadt, Germany; 4 Private Practice for Education, Consultation and Therapy, Amsterdam, The Netherland; 5 Marie Cederschiölds University, Stockholm, Sweden; 6 TF-CBT Australia, Sydney, NSW, Australia; 7 Department of Psychiatry, Allegheny General Hospital, Pittsburgh, PA, USA; 8 Rowan-Virtua School of Osteopathic Medicine, CARES Institute, Stratford, NJ, USA; 9 Department of Psychiatry, Rowan-Virtua School of Osteopathic Medicine, Stratford, NJ, USA; 10 Private Practice, Augsburg, Germany; 11 Chan Medical School, Department of Psychiatry, University of Massachusetts, MA, USA; 12 Private Practice, Enhetshälsa Sverige AB, Ängelholm Sweden; 13 Child and Youth Psychiatry, Falun Sweden, BUP-Capio, Falun, Sweden; 14 Private Practice for Education and Consultation, Psykolog Onsjö, Gothenburg, Sweden; 15 Private Practice, Oslo, Norway; 16 Psychology Department, University of Oslo, Oslo, Norway; 17 Centre for Support and Treatment, Save the Children Sweden, Göteborg, Sweden; 18 Centre for Support and Treatment, Save the Children Sweden, Stockholm, Sweden; 19 University of Luxemourg, Esch-sur-Alzette, Luxembourg; 20 Mental Health for Ukraine Project, Lviv City, Ukraine; 21 Department of Clinical Child and Adolescent Psychology, Otto Friedrich University Bamberg, Bamberg, Germany

**Keywords:** children, trauma-focused treatment, Ukraine, war, PTSD

## Abstract

**Background:**

The large-scale Russian invasion of Ukraine in early 2022 resulted in a humanitarian crisis with hundreds of thousands of children exposed to traumatic events. To date, trauma-focused evidence-based treatments (EBTs) for children and youth have not been systematically evaluated and implemented in Ukraine. This study aims at evaluating 1) the feasibility of a training program for Ukrainian therapists on Trauma-Focused Cognitive Behavioural Therapy (TF-CBT) and 2) the feasibility and effectiveness of the treatment for children, youth, and their families in and from Ukraine during the ongoing war.

**Methods:**

The project “TF-CBT Ukraine” was implemented between March 2022 and May 2024, in close collaboration with local and international partners. Therapists completed questionnaires before/after the training, and patients were asked to complete a measure on PTSD before and after treatment.

**Results:**

Altogether 138 therapists started the training program and 44.9% were certified as TF-CBT therapists. The program completers reported overall high satisfaction with the training program, a positive change in their attitude towards EBTs and trauma-related knowledge gain. The patients (age 3–21, 37% male) reported significant improvement in symptoms of PTSD at the end of treatment with large pre-post effect sizes for DSM-5 PTSD (*d*_selfreport_ = 2.36; *d*_caregiverreport_ = 2.27), ICD-11 PTSD (*d*_selfreport_ = 1.97; *d*_caregiverreport_ = 1.77), ICD-11 CPTSD (*d*_selfreport_ = 2.04; *d*_caregiverreport_ = 1.99), and DSM-5 pre-school PTSD (*d_caregiverreport_* = 3.14).

**Conclusions:**

The results of this study are promising in regard to the general implementation of trauma-focused EBTs in active conflict areas. Future studies need to replicate these findings in a randomized controlled study design.

## Introduction

The Russian Invasion of Ukraine, which started in February 2022, is a dramatic escalation of the armed conflict since 2014. This war places an entire generation of Ukrainian children under severe strain. Preliminary epidemiological studies on the atrocities these children and adolescents experience and the mental health problems they may develop thereafter are still scarce, and potential long-term consequences are unknown. Recent studies before and during the invasion with Ukrainian children and adolescents indicate high prevalence rates of traumatic events (war-related and pre-war trauma) as well as trauma-related disorders such as posttraumatic stress disorder (PTSD), anxiety, or depression [1–3].

The implementation and evaluation of efficient treatment protocols for children impacted by traumatic events in Ukraine are therefore of utmost importance. Given the extremely high number of children and adolescents who may suffer from a trauma-related mental health disorder due to the war and earlier traumatic experiences [4] on the one hand and the lack of evidence-based trauma-focused treatments in Ukraine on the other hand [5], (inter-)national efforts are needed to fill this gap in the current mental health care services in Ukraine during the war [6]. There is a large evidence base for effective trauma-focused individual treatment protocols for children and adolescents [7], but very little evidence on their feasibility and effectiveness for children who are living under a current threat, such as an ongoing war. An earlier study by Cohen et al. [8], however, offers guidance in how to tailor such treatment for children who suffer from ongoing trauma. Common sense among experts and practitioners is actually rather to not offer such treatment during the current threat, but instead after the patient is under “safe living circumstances.” Given the uncertainty of how long this war might continue and the fact that posttraumatic stress symptoms (PTSS) can become a severely harmful chronic condition which has long-term consequences for the child’s development if left untreated [9, 10], initiatives designed to offer and evaluate such treatment for children in the context of active war conditions are important.

Together with a large group of Ukrainian and international experts, funders, and stakeholders, the project TF-CBT Ukraine [11] was developed right after the beginning of the large-scale invasion in March 2022. This study aims to evaluate the feasibility of a comprehensive TF-CBT training program during an ongoing war and to evaluate the feasibility and effectiveness (uncontrolled design) of the TF-CBT treatment in a war-torn country like Ukraine.

## Methods

Please see [11] for a detailed description of the overall study design and methods. Among the collaboration partners and funders are the TF-CBT treatment developers, certified international TF-CBT trainers, the National Psychological Association of Ukraine, the National Child Traumatic Stress Network (USA), the Ministry of Health of Ukraine and Ministry of Education and Science of Ukraine and the “Mental health for Ukraine Project,” implemented by GFA Consulting Group GmbH, the EMDR Europe Association, the Porticus Foundation, and the CARES Institute at Rowan Medicine in the US. The project received ethical approval by Ulm University (Number: C1/Sta) in Germany and the Zhytomyr Ivan Franko State University (Number: 9–08072022) in Ukraine. All therapists gave their written informed consent prior to study inclusion. The project started in March 2022 and officially ended in May 2024. On a sidenote, within this project, data were collected on the efficacy of EMDR treatment as well; results will be described in a separate publication.

### Recruitment and participants

Ukrainian therapists were informed about the project via information leaflets posted in social media channels by our Ukrainian partners. We applied the following inclusion criteria for Ukrainian therapists: 1) be a mental health care professional in or from Ukraine; 2) basic knowledge of CBT principles; 3) willingness to participate in the full program. Besides age, there were no criteria for patients who could be treated by the therapists, but during the basic training, the therapists received information on who might benefit best from the treatment (e.g. trauma history, elevated PTSS).

### TF-CBT training program

The training program entailed the following consecutive steps for therapists: 1) web-based training [12] or reading of the Ukrainian/Russian TF-CBT treatment manual; 2) participation in a virtual three-day basic training on TF-CBT; 3) participation in at least 10 out of 12 monthly case consultation calls; 4) assessment and treatment of at least 3 patients during one year after the basic training. If the therapist completed all training steps, he/she received a TF-CBT therapist certificate. Additionally, the therapists could participate in several extra sessions on related topics that arose during the program. The following sessions were offered: traumatic grief, trauma assessment, related measurements in the field of trauma treatment, caregiver involvement, strategies of implementing TF-CBT during ongoing trauma exposure, sexual development and sexually problematic behavior in children and adolescents, treatment of depression in a trauma context, and suicidality. Finally, all therapists were invited to a PRACTICE skills course to support their professional and personal well-being [13]. All program components were delivered by certified international TF-CBT trainers (basic training and case consultations, extra sessions) and international experts in the field of child/adolescent mental health (extra sessions). Simultaneous translations of the training components and translated therapy and assessment materials were provided.

### TF-CBT intervention

TF-CBT is a short-term (12–16 weekly, 60–90-minute parallel or conjoint sessions with caregivers), component-based EBP for children and youth impacted by trauma [14]. TF-CBT integrates cognitive, behavioral, interpersonal, and family therapy principles and consists of three treatment phases: stabilization and skills building (sessions 1–4), trauma narration and processing (sessions 5–8), and integration (fostering safety and future development; sessions 9–12).

### Measures

The therapists completed questionnaires before their training participation (T0) and after having completed all training steps, just before they received their certificate (T1). Additionally, they were asked to complete the TF-CBT Brief Practice Fidelity Checklist (BPCL TF-CBT) [15] for each patient during the treatment. The patients and their caregivers were asked to complete questionnaires before (F0) and after treatment (F1).

#### Training program implementation and evaluation

Via extensive study monitoring, we collected data on participation numbers of all training components and extra sessions across all training cohorts. The implementation was regularly evaluated by the therapists via brief surveys after each component with several open questions, and one question on overall satisfaction (0 = *very dissatisfied* to 10 = *very satisfied*). General satisfaction with the overall training program and a general program evaluation was assessed from all therapists who completed all training components in the T1 survey via 13 closed and 4 open unstandardized items (see Table 1 for more details).

**Table 1.** Program evaluation and satisfaction of the therapists assessed at post-training (T1)

	M (SD)
*Please rate the quality of…* ^a^	4.79 (.45)
the TF-CBT training program.	4.78 (.45)
the 3-day TF-CBT basic training.	4.80 (.44)
the case consultations.	4.74 (.48)
the optional sessions	4.79 (.45)
How much did you learn about trauma-focused treatments for traumatized children and adolescents?^b^	4.00 (.00)
Did you have any difficulties in implementing the treatment?^c^	2.44 (.56)
Do you plan on treating more patients with TF-CBT after the training program?^d^ *n(%)*	65 (98.48)
*Aims and satisfaction*^e^	
I have acquired skills to be able to help traumatized children and adolescents	1.08 (.27)
I have satisfactorily overcome difficulties in the implementation of the sessions	1.32 (.64)
I was able to deal with strong feelings of the participants	1.29 (.49)
I worked on optimizing the care of traumatized children and adolescents in/from Ukraine	1.23 (.49)
Overall, I have become more sensitive to traumatic events and trauma-related disorders	2.08 (1.15)
I am satisfied with my performance in the therapy sessions	1.52 (.53)
I am satisfied with my participation in the project	1.12 (.37)

a 1 = not good at all; 2 = ok; 3 = good; 4 = very good; 5 = great;

b 1 = Nothing; 2 = a little bit; 3 = some things; 4 = a lot;

c 1 = none; 2 = a few; 3 = moderate; 4 = a lot; 5 = almost every session;

d 1 = Yes;

e 1 = yes; 2 = rather yes; 3 = Partly; 4 = Rather no; 5 = no.

#### Therapist assessment

*Socio-demographic* information such as age, gender, current location at the beginning of the training program, the professional background, and experience with the treatment of PTSD is assessed via an unstandardized questionnaire.

*The Professional Quality of Life scale* (ProQoL) [16] assesses via 30 items on a 5-point Likert scale (1 = *never* to 5 = *very often*) the following three subscales of professional quality of life: (1) compassion satisfaction, (2) burnout and (3) secondary traumatic stress (STS) of professionals. For each subscale, 10 items are summed up. Sum scores less than 23 indicate low values for compassion satisfaction, burnout, or STS. Sum scores between 23 and 41 indicate moderate values for the three subscales, and sum scores above 41 indicate high values for the corresponding subscales. In the present study, the subscales have shown questionable to excellent reliability (*α*_pre_ = .67–.82; *α*_post_ = .67–.90).

The *Evidence-Based Practice Attitude Scale* (EBPAS-36; [17] is a 36-item questionnaire, assessing the attitudes towards the adoption of evidence-based practice on a 5-point Likert-scale (0 = *not at all* to 4 = *very great extent*). The scale distinguishes 12 dimensions of three items each: (1) appeal, (2) requirements, (3) openness, (4) divergence, (5) limitations, (6) fit, (7) monitoring, (8) balance, (9) burden, (10) job security, (11) organizational support, (12) feedback. A detailed explanation of the subscales can be found elsewhere [17]. A total scale score representing the global attitude towards EBPs is calculated by adding the sum scores of the subscales. Higher values indicate a more positive attitude towards EBPs. The EBPAS-36 has been well validated [17, 18] and in the present study has shown good reliability for the total scale (*α*_pre_ = .87; *α*_post_ = .85).

*Trauma-related knowledge* is assessed via six unstandardized questions. The perceived importance of trauma confrontation/ exposure, the therapeutic relationship, and evidence-based therapies is measured on a 5-point Likert-scale (1 = *very important* to 5 = *not important at all*). Additionally, the self-evaluated knowledge of traumatic events, trauma-related disorders, and therapeutic methods for trauma-related disorders is measured on a 5-point Likert-scale (1 = *no knowledge* to 5 = *very high*) at T1.

#### Treatment fidelity and effectiveness

Treatment fidelity was assessed by a modified version of the TF-CBT Brief Practice Fidelity Checklist (BPCL TF-CBT) [15]. Therapists indicated which of the nine PRACTICE components they implemented during the treatment with the patient (0 = *fidelity is not met*, 1 = *fidelity is met*).

#### Patient assessment

The *Child and Adolescent Trauma Screen* Second Version (CATS-2) [19] measures potentially traumatic events (PTEs) and posttraumatic stress symptoms (PTSS) according to DSM-5 and ICD-11 criteria for children and adolescents from 7 to 21 years old (self- and caregiver version). First, the experience of PTEs is assessed via a 15-item structured PTE checklist. Subsequently, PTSS in the last 4 weeks is assessed by 25 items rated on a 4-point Likert scale (0 = *Never*, 1 = *Sometimes*, 2 = *Often*, 3 = *Almost Always*). The sum of all items (range 0–60), forms the DSM-5 PTSD severity score, whereas the ICD-11 PTSD severity score is the sum of 6 items (range 0–18). The ICD-11 CPTSD score (range 0–36) is the sum of the ICD-11 PTSD severity score plus the sum of the ICD-11 DSO severity score (6 items).

In the current study, the measure had a questionable to good reliability (*α*_DSM-5 PTSD_ = .87; *α*_ICD-11 PTSD_ = .68; *α*_ICD-11 CPTSD_ = .81). In addition to the self-report measure for children and adolescents, the caregivers were asked to fill out a parallel caregiver version, which showed a good to excellent reliability (*α*_DSM-5 PTSD_ = .90; *α*_ICD-11 PTSD_ = .80; *α*_ICD-11 CPTSD_ = .85).

The CATS preschool version for children aged between 3 and 6 years [20] was implemented to assess preschool children. The questionnaire comprises a 15-item PTE checklist and a PTSS symptom checklist in the last two weeks, with 16 items rated on a 4-point Likert scale (0 = *Never*, 1 = *Sometimes*, 2 = *Often*, 3 = *Almost Always*). The total symptom score was calculated by summing up all items (range 0–48). The reliability of the caregiver version of the CATS in the current study was acceptable (*α*_DSM-5 PTSD_ = .79).

Following the diagnostic algorithms of the DSM-5 and ICD-11, the categorical item-mapping approach of the CATS-2 was followed [19], with a symptom being rated as present for values of 2 = *Often* or 3 = *Almost Always.*

### Statistical methods

Analysis was performed using IBM SPSS Statistics version 29. All tests were two-tailed, and an alpha level of *p* < .05 was used.

#### Therapist outcomes

Descriptive analyses were performed to profile the sociodemographics of the sample. To explore changes in study variables between T0 and T1, unpaired *t*-tests were computed.

#### Patient outcomes

Main analyses followed intention to treat principles, including all patients undergoing F0 CATS-2 screening, irrespective of dropout, treatment dose, or missing F1 data. The rates of missing data at F1 were 27.70% [81 of 292] for the 7–21 years sample and 16.31% [5 of 31] for the 3–6 years sample. No differences for baseline (F0) characteristics such as age, gender, trauma load, and CATS F0 scores were found between participants with or without missing data. Only in the 3–6 years old sample, there was a significant difference between patients with and without missing data at F1 regarding the DSM-5 total score at F0 (*M*_missing_ = 24.80; *M*_completer_ = 32.62; *t*(29) = 2.36. *p* = .025). Two missing value analyses indicated that Little’s test of Missing Completely at Random (MCAR) was not significant for the 3–6 years sample (χ^2^ (1) = 1.10, *p* = .294) and for the 7–21 years sample (χ^2^ (3) = 5.49, *p* = .140).

Mixed effect models, with fixed effects of time and fixed effects of the covariates age, gender, and location of the child (inside Ukraine vs. other countries), as well as the time × gender, time × age, and time × location interactions were performed on all CATS-2 scales. Random effects were not included in the final models as this worsened the likelihood criteria of the models. Based on the longitudinal design of the study, data were nested by participants and repeated measures were modeled using an unstructured covariance matrix based on the comparison of likelihood criteria of model fit (AIC and BIC). Mixed effect models can handle missing data under the missing at random assumption. Parameters were estimated using the restricted maximum likelihood (REML) method.

## Results

The therapist sample (who completed T0 assessment) comprised *N* = 138 therapists (97.10% female; *M*(age) = 39.59 (*SD* = 8.82; range 22–65)) who were mostly located in Ukraine (86.23%). Most were psychologists (88.41%) and/ or psychotherapists (20.29%). They reported an average of 7.55 years of therapeutic experience (*SD* = 6.37; range 0–31). The majority of the therapists (*n* = 88; 63.8%) reported a therapeutic background in CBT. Other therapeutic backgrounds of the participants were DBT (*n* = 18; 11.7%), Analytic (*n* = 7, 5.1%), Psychodynamic (*n* = 16; 11.7%) or other (*n* = 42, 30.4%). At T1, *n* = 66 (47.83%) therapists completed the final survey.

The patient sample comprised *N* = 327 children, adolescents, and young adults. Of those, *n* = 4 were older than 21 years and were excluded from the present study. Thus, the final sample consisted of *n* = 323 patients (*n* = 200, 61.92% female) with a mean age of 12.22 years old (*SD* = 4.02; range 3–21 years), 9.60% (*n* = 31) were pre-school age and 4.95% (*n* = 16) were older than 17 years old. More than half of the patients (*n* = 216, 66.87%) were, at the time of F0 assessment, still located in Ukraine. Regarding the other patients, *n* = 1 reported to live in the occupied territory of Ukraine, and the other patients resided in 19 different countries, primarily in Europe. The most common countries were Germany (*n* = 31, 9.4% of all patients), the UK (*n* = 9, 2.7%), and Poland (*n* = 8, 2.4%). Please see Pfeiffer, Garbade, and Sachser [11] for more information on the cross-sectional data of the patient sample.

### Training program implementation and evaluation

Altogether, *N* = 243 therapists signed up for the program across all nine cohorts, with 5–30 participants in each cohort (*M* = 14.44; *SD* = 7.92). Subsequently, *n* = 138 (56.80%) actually started with the program and completed the web-training/ read the manual, *n* = 130 went on to participate in the basic training. Only the therapists who participated in the basic training were invited to the case consultations and started treating patients with the model. Altogether, *n* = 73 therapists attended at least 10 case consultations, and *n* = 67 treated at least 3 patients with TF-CBT. Finally, *n* = 62 completed all steps and were officially certified as TF-CBT therapists (47.69% of those who completed the basic training and 25.51% who initially registered to participate). Participation rates in the extra sessions were between 20–35 participants per session (*M* = 27.14; *SD* = 5.05).

In the regular feedback surveys, the satisfaction ratings with the basic trainings (*M* = 9.25; *SD* = 1.24; range 4–10) and extra sessions (*M* = 9.10; *SD* = 1.56; range 0–10) were high. Please see Table 1 for evaluation and satisfaction ratings of the therapists at T1. All participants indicated that they had learnt “a lot” about trauma-focused treatments for children and adolescents impacted by traumatic experiences. On average, they rate their knowledge on traumatic events for children and adolescents, trauma-related disorders, and therapeutic methods for trauma-related disorders as high.

Most of the therapists evaluated the quality of a) the TF-CBT training program, b) the 3-day TF-CBT basic training, c) the case consultations, and d) the optional sessions as great. Most therapists reported difficulties implementing TF-CBT, which may not be surprising given the active war conditions therapists and clients were enduring. Their training aims were overall fulfilled, and therapists were on average highly satisfied with the training.

Please see Table 2 for changes in ProQol, EBPAS, and knowledge-related questions. Compassion satisfaction of participating therapists increased significantly. There were no statistically significant changes in regard to burnout and STS from T0 to T1. Attitudes towards EBP significantly improved, and trauma-related knowledge increased from T0 to T1, with significant changes in the perceived importance of the therapeutic relationship (Table 2).

**Table 2.** Descriptive data of therapists’ professional quality of life, attitudes towards evidence-based treatments and trauma-related knowledge before and after the training program

	Before training (T0) M (SD)	After training (T1) M (SD)	*t*(202)	*p*	Cohen’s *d*
*Professional quality of life*					
Compassion satisfaction	40.64 (4.12)	42.80 (3.99)	−3.539***	<.001	.530
Burnout	19.71 (3.69)	19.29 (3.56)	.773	.440	.178
Secondary traumatic stress	19.61 (4.31)	18.97 (3.44)	1.054	.293	.136
*Attitudes towards evidence-based treatment*					
Total	3.87 (.40)	3.99 (.36)	−2.069*	.040	.310
Requirement	3.26 (.94)	3.08 (.93)	1.320	.188	.198
Appeal	4.04 (.68)	4.16 (.55)	−1.254	.211	.188
Openness	4.09 (.67)	4.26 (.57)	−1.763	.079	.264
Divergence	2.11 (.77)	1.98 (.64)	1.217	.225	.182
Limitations	1.81 (.74)	1.55 (.68)	2.422*	.016	.362
Fit	4.27 (.62)	4.48 (.50)	−2.452*	.015	.367
Monitoring	2.31 (.98)	1.95 (.95)	2.454*	.015	.367
Balance	2.90 (.74)	3.01 (.80)	−.937	.350	.140
Burden	1.42 (.52)	1.39 (.49)	.382	.703	.057
Job security	3.25 (.98)	3.43 (1.02)	−1.231	.110	.184
Organizational support	3.70 (.95)	3.80 (1.03)	−.668	.505	.100
Feedback	4.34 (.62)	4.50 (.59)	−1.743	.083	.261
*Trauma related knowledge*					
Importance confrontation/exposure	1.68 (.83)	1.53 (.66)	1.295	.197	.194
Importance therapeutic relationships when treating PTSD	1.25 (.47)	1.08 (.27)	2.769**	.006	.414
Importance evidence-based therapies	1.28 (.50)	1.26 (.48)	0.243	.808	.036

*Note*: Bold values indicate that they are statistically significant.

* *p* < .05;

** *p* < .01;

*** *p* < .001.

### Treatment fidelity

At T1, therapists indicated that they had implemented *M* = 7.54 (*SD* = 1.83) of the nine PRACTICE components. The most frequently applied components were “Psychoeducation” (*n* = 244, 92.78%) and “Parenting skills” (*n* = 244, 92.78%). The most rarely applied component was “In-vivo desensitization” (*n* = 135, 51.14%). The trauma narration and processing component was applied in 87.83% cases (7–21 years: 86.50% and 3–6 years: 100.00%). See Table 3 for more details.

**Table 3.** Fidelity checklist.

	Total sample (*n* = 263) (*n*, %)	7–21 years (*n* = 237) (*n*, %)	3–6 years (*n* = 26) (*n*, %)
Psychoeducation	244 (92.78)	218 (91.98)	26 (100.00)
Parenting skills	244 (92.78)	218 (91.98)	26 (100.00)
Relaxation skills	206 (78.33)	180 (75.95)	26 (100.00)
Affective regulation	234 (88.97)	211 (89.03)	23 (88.46)
Cognitive coping	224 (85.17)	203 (85.65)	21 (80.77)
Trauma narrative	231 (87.83)	205 (86.50)	26 (100.00)
In-vivo desensitization	135 (51.33)	116 (48.95)	19 (73.08)
Conjoint youth-parent sessions	230 (87.45)	204 (86.08)	26 (100.00)
Enhancing safety	236 (89.73)	211 (89.03)	25 (96.15)

### Patient outcomes

The participants aged 7 years and older reported on average 4.64 different PTEs (*SD* = 2.68, range 0–13). The most frequently reported PTEs were “war” (*n =* 202; 68.94%), “bullying” (*n* = 139, 47.44%), and “witnessing domestic violence” (*n* = 122, 41.64%). For preschool children, the caregivers reported an average of 4.45 PTEs (*SD* = 2.45, range 1–10). The most frequently reported PTEs were “war” (*n* = 22, 71.97%), “witnessing a violent attack” (*n* = 15, 48.39%), and “witnessing family violence” (*n* = 13, 41.94%). For more information on the reported PTEs, please see Additional file 1: Supplementary Tables S1 and S2. Of the participants aged 7 years and older, *n* = 196 (66.2%) lived in Ukraine and *n* = 89 (30.1%) outside of Ukraine. Of the preschool children, *n* = 22 (71.0%) lived in Ukraine, and *n* = 9 lived outside of Ukraine.

Estimated means and standard deviations of the CATS scores based on the linear mixed effect models are depicted in Table 4. Categorical analyses of the PTSS symptoms revealed that *n* = 210 (71.67%) of participants aged 7 years and older (self-report) fulfilled all clinical criteria for a PTSD diagnosis according to DSM-5 at F0 and *n* = 8 (3.38%) at F1. According to ICD-11, *n* = 90 (30.71%) fulfilled the criteria for a PTSD diagnosis, and *n* = 70 (23.89%) fulfilled the criteria for a CPTSD diagnosis at F0. At F1, *n =* 3 (1.27%) fulfilled the ICD-11 criteria for a PTSD diagnosis, and *n =* 2 (0.84%) for a CPTSD diagnosis. For preschool children, *n* = 28 (90.32%) fulfilled the criteria for a PTSD diagnosis according to DSM-5 at F0 and *n =* 0 (0.00%) at F1. For both age groups, linear mixed models showed a significant main effect of time for all PTSD severity scores using self- and caregiver-reports, indicating statistically significant improvement of PTSS symptoms during TF-CBT treatment (Table 5), with large pre-post effect sizes for DSM-5 PTSD (*d*_selfreport_ = 2.36; *d*_caregiverreport_ = 2.27), ICD-11 PTSD (*d*_selfreport_ = 1.97; *d*_caregiverreport_ = 1.77), ICD-11 CPTSD (*d*_selfreport_ = 2.04; *d*_caregiverreport_ = 1.99), and DSM-5 pre-school PTSD (*d* = 3.14). Interaction effects for gender and age were statistically non-significant, besides a significant time × age interaction for self- and caregiver-reported CPTSD symptoms in the 7–21 years sample, indicating a higher improvement for older youth in this sample regarding CPTSD symptoms. Regarding location (child inside Ukraine vs. outside Ukraine) we found significant main effects for all reported self and caregiver scales (DSM-5 PTSD, ICD-11 PTSD and ICD-11 CPTSD), indicating significantly higher PTSS in children outside Ukraine. However, these differences were clinically not meaningful (estimated 0.32–1.92 mean point differences on the scales). The time x location interaction was significant for the self-report of ICD-11 PTSD and CPTSD, indicating higher improvement for children and adolescents living in Ukraine. However, these differences were clinically not meaningful. In the pre-school sample, neither a significant main effect of location nor a time × location interaction emerged.

**Table 4.** Estimated means and standard deviations from the linear mixed effect models

	Estimates before treatment (F0) (*M*, (*SE*))	Estimated after treatment (F1) (*M*, (*SE*))	Statistics
*Self-report (7–21 age)*			
CATS–2 DSM–5 PTSD	37.54 (0.71)	12.13 (0.58)	*p* < .001
CATS–2 ICD–11 PTSD	10.79 (0.26)	3.25 (0.19)	*p* < .001
CATS–2 ICD–11 CPTSD	20.48 (0.48)	6.24 (0.35)	*p* < .001
*Caregiver-report (7–21 age)*			
CATS–2 DSM–5 PTSD (caregiver 7–21)	36.89 (0.84)	10.92 (0.57)	*p* < .001
CATS–2 ICD–11 PTSD (caregiver 7–21)	10.07 (0.32)	2.79 (0.19)	*p* < .001
CATS–2 ICD–11 CPTSD (caregiver 7–21)	19.85 (0.55)	5.56 (0.32)	*p* < .001
*Caregiver-report (3–6 age)*			
CATS DSM–5 PTSD	31.32 (1.54)	7.95 (1.10)	*p* < .001

**Table 5.** Linear mixed-effect models for PTSS change in patients

Model	Fixed effect	Estimate (*B*)	*SE*	*df*	*T*	Sig.	95% CI
*7–21 years self-report*							
DSM–5 PTSD	Intercept	12.38	2.24	227.91	5.54	< .001	[7.97; 16.78]
	Time	19.14	3.03	263.10	6.32	< .001	[13.18; 25.10]
	Gender	0.77	1.09	226.27	0.71	.481	[−1.38; 2.91]
	Location	−2.52	1.10	226.58	−2.28	.024	[−4.69; −0.34]
	Age	0.05	0.15	226.84	0.32	.753	[−0.25; 0.35]
	Time × gender	0.37	1.50	253.08	0.25	.806	[−2.59; 3.33]
	Time × location	2.38	1.52	254.77	1.57	.118	[−0.61; 5.38]
	Time × age	0.38	0.21	256.25	1.79	.075	[−0.04; 0.79]
ICD–11 PTSD	Intercept	3.60	0.74	229.61	4.90	< .001	[2.15; 5.05]
	Time	6.31	1.06	269.50	5.94	< .001	[4.22; 8.40]
	Gender	0.11	0.36	227.74	0.29	.770	[−0.60; 0.81]
	Location	−0.94	0.36	228.09	−2.59	.010	[−1.66; −0.23]
	Age	0.01	0.05	228.38	0.09	.928	[−0.10; 0.10]
	Time × gender	−0.00	0.53	259.22	−0.01	.996	[−1.04; 1.04]
	Time × location	1.23	0.53	260.93	2.31	0.22	[0.18; 2.29]
	Time × age	0.05	0.07	262.44	0.64	.52	[−0.10; 0.19]
ICD–11 CPTSD	Intercept	6.29	1.35	232.51	4.65	< .001	[3.63; 8.95]
	Time	9.04	1.90	274.63	4.75	< .001	[5.29; 12.79]
	Gender	0.35	0.66	230.45	0.53	.596	[−0.95; 1.65]
	Location	−1.79	0.67	230.83	−2.68	.008	[−3.11; −0.48]
	Age	0.05	0.09	231.16	0.56	.576	[−0.13; 0.23]
	Time × gender	0.38	0.95	264.40	0.41	.686	[−1.48; 2.25]
	Time × location	2.06	0.96	266.11	2.15	.032	[0.18; 3.95]
	Time × age	0.31	0.13	267.62	2.31	.022	[0.05; 0.57]
*7–21 years caregiver-report*							
DSM–5 PTSD	Intercept	12.42	2.21	202.97	5.62	< .001	[8.06; 16.78]
	Time	22.33	3.44	241.89	6.48	< .001	[15.55; 29.12]
	Gender	0.56	1.07	201.91	0.52	.602	[−1.55; 2.66]
	Location	−2.28	1.10	201.99	−2.08	.039	[−4.44; −0.12]
	Age	−0.05	0.16	202.55	−0.32	.749	[−0.37; 0.26]
	Time × gender	−1.61	1.71	233.10	−0.94	.347	[−4.98; 1.76]
	Time × location	0.71	1.76	234.78	0.41	.685	[−2.75; 4.17]
	Time × age	0.32	0.25	238.39	1.29	.198	[−0.17; 0.82]
ICD–11 PTSD	Intercept	3.73	0.71	208.88	5.22	< .001	[2.32; 5.14]
	Time	6.11	1.17	248.10	5.20	< .001	[3.79; 8.42]
	Gender	0.17	0.35	206.67	0.49	.627	[−0.51; 0.85]
	Location	−1.00	0.36	206.82	−2.82	.005	[−1.70; −0.30]
	Age	−0.04	0.05	207.99	−0.81	.419	[−0.14; 0.06]
	Time × gender	−0.67	0.58	239.57	−1.15	.251	[−1.82; 0.48]
	Time × location	0.62	0.60	240.75	1.03	.302	[−0.56; 1.80]
	Time × age	0.10	0.09	244.54	1.11	.267	[−0.07; 0.26]
ICD–11 CPTSD	Intercept	6.10	1.26	205.62	4.86	< .001	[3.63; 8.58]
	Time	9.78	2.12	248.57	4.62	< .001	[5.62; 13.95]
	Gender	0.18	0.61	204.19	0.30	.762	[−1.01; 1.38]
	Location	−1.42	0.62	204.29	−2.28	.023	[−2.65; −0.19]
	Age	0.01	0.09	205.05	0.07	.948	[−0.17; 0.18]
	Time × gender	−0.22	1.05	240.09	−0.21	.837	[−2.29; 1.86]
	Time × location	0.84	1.08	241.42	0.78	.437	[−1.29; 2.97]
	Time × age	0.33	0.15	245.07	2.15	.032	[0.03; 0.64]
*3–6 years caregiver-report*							
DSM–5 PTSD	Intercept	6.79	6.40	22.89	1.07	.297	[−6.36; 19.94]
	Time	31.20	12.20	27.33	2.56	.016	[6.17; 56.22]
	Gender	−1.58	2.20	22.96	−0.72	.481	[−6.13; 2.97]
	Location	2.89	2.25	22.79	1.29	.212	[−1.77; 7.55]
	Age	0.10	1.15	22.84	0.09	.933	[−2.28; 2.48]
	Time × gender	−0.31	4.17	27.72	−0.07	.942	[−8.84; 8.23]
	Time × location	−2.96	4.39	26.91	−0.67	.506	[−11.97; 6.05]
	Time × age	−1.21	2.23	27.13	−0.54	.592	[−5.77; 3.36]

## Discussion

This is the first study that implemented and evaluated a training program for an individual evidence-based trauma-focused treatment for children and adolescents in active war in Ukraine. Recruitment and attendance rates of therapists were surprisingly high, given their current living circumstances. A certification rate of almost 50% for those who attended the basic training, as well as their high satisfaction and quality ratings of training components, indicate high acceptance of the training program among Ukrainian therapists in war circumstances. However, almost all therapists reported difficulties in implementing the treatment (e.g. relocation of clients due to war, parents first need treatment themselves, fear of stigmatization), which may reflect the challenges of providing trauma-focused treatment in active war conditions and many therapists not being used to EBT and CBT protocols. In addition, the difficulties reported by therapists may highlight the importance of flexibility in implementing the model on the one hand, and the necessity of continuous support (e.g. case consultations and extra sessions on relevant topics that hinder treatment delivery) on the other hand. Rates of Compassion Satisfaction significantly increased, but not levels of Burnout and STS, which could be explained by low rates at baseline. Compared to similar therapist samples who do not experience war during the study [21], rates of burnout and STS were comparably low, which indicates a humbling resilience in this population [22, 23]. Nevertheless, it is important to keep in mind that the therapists might experience trauma themselves and face difficult living circumstances which is why self-care programs for therapists should be an important part of such training programs [24]. The therapists’ attitudes towards EBTs were already rather high at baseline, but increased during program implementation, and therapists reported knowledge gain regarding trauma-related aspects. These results are promising for the feasibility and effectiveness of training programs in trauma-focused EBTs in a country that is at war and in which EBTs are normally not implemented.

Regarding the effectiveness of the treatment itself, results showed significant PTSS reductions from baseline to post-treatment across all criteria (DSM-5, ICD11 PTSD, and CPTSD) and age groups, with large effect sizes, independent of age and gender (besides CPTSD self-report). Inspecting a possible differential effect on children and adolescents living inside Ukraine compared with children and adolescents living outside of Ukraine revealed non-significant differences for some scales (all caregiver-reports and self-reported DSM-5 PTSD) and higher improvements for youth inside Ukraine for ICD-11 PTSD and CPTSD. As these differences were clinically not meaningful, this effect should not be overinterpreted. Overall, the PTSS symptom change effect sizes are higher compared with other TF-CBT studies [25] and other trauma-focused EBTs for children [26], which could, to some extent, be explained by the non-controlled design. The categorical analysis further showed high effectiveness in regard to the prevalence of patients who did not fulfil PTSD criteria post-treatment. Interestingly, next to war, the patients reported high rates of other PTEs, such as domestic/community violence or bullying. Hence, next to war trauma, many other PTEs might have been addressed in TF-CBT treatment. In regards to feasibility, the session checklists revealed that fidelity was similar or slightly lower compared to other RCTs [27] and naturalistic studies on TF-CBT [28] with therapists delivering on average 7.5 of 9 PRACTICE components and 88.00% delivering trauma narration and processing. Given the non-controlled design in this study and the focus on dissemination instead of high internal validity, the fidelity can be perceived as acceptable. Non-completer/drop-out rates (no F1 data in 19.17% 7–21 years old, 16.12% preschool children) were slightly higher compared to other individual trauma-focused EBTs (10.6–15.5%) [29], but lower compared to regular outpatient treatment [30]. Given the war circumstances, these low drop-out rates could be considered promising, as therapists frequently reported in case consultations that either they or their patients were often forced to change their location.

### Limitations

The TF-CBT Ukraine project aimed at training as many therapists as possible and making TF-CBT available to as many children as possible – which means that there was a stronger focus on dissemination than on evaluation. Given this premise, we also tried not to overload therapists and patients with assessments, although many other aspects would have been interesting to better understand different aspects of the feasibility and effectiveness. For example, children and adolescents who experience PTEs may develop many other trauma-related disorders in addition to PTSD [31], which should be assessed in future similar projects. Moreover, parents’ (or other primary caregivers) trauma-related symptomatology is known to have an impact on their child’s symptomatology [32], this could be especially influential given that they also experience war. Hence, future studies should assess and monitor the parents’ responses to TF-CBT as well. Furthermore, we did not systematically assess whether treatments were delivered in an online format or the reasons for training interruptions, which could offer valuable insights for future implementation efforts. Although the efficacy of TF-CBT has been demonstrated in more than 20 RCTS [25], an RCT design to better understand the high treatment gains and potential other contributing factors in children’s recovery during war conditions might be beneficial. Due to the anonymity of the data, it is also not possible to investigate potential therapist-related moderators of the outcome, such as the CBT background of the therapists. Regarding study methods, it is noteworthy that the subscales of the EBPAS and ProQol showed low internal validity, the measures were only translated forward (not backwards), and we could not match all T0 and T1 data. Lastly, the therapists were asked to assess and submit the patient data, which might limit the validity of the data. Thus, due to the anonymous online assessment, double entries of patient and therapist data cannot be ruled out. Moreover, in this study, we only report the data of patients that was submitted by the therapists for them to receive their certificate. We do expect them to have treated many more patients, though.

## Conclusion

This is the first study that evaluate the efficacy and feasibility of TF-CBT in a conflict setting. As both the results on the patient and therapist levels are promising, this collaborative initiative hopefully facilitates future funding and infrastructure for TF-CBT and other EBPs to be implemented in conflict settings. The digital implementation of the training program, combined with simultaneous translation, could enable training opportunities in other countries in which there are no local trainers in the respective EBTs. The positive results on effectiveness and feasibility of the treatment contribute to the ongoing discussion on delivering trauma-focused EBTs during ongoing trauma and shed light on the importance of offering such treatment and training to a population that experiences trauma on a societal scale. This project demonstrated the high value of local as well as international partnerships in these challenging circumstances.

## Supporting information

10.1192/j.eurpsy.2025.10032.sm001 Pfeiffer et al. supplementary material Pfeiffer et al. supplementary material

## Data Availability

The data is available from the authors upon request.

## Abbreviations

BPCL TF-CBT: TF-CBT Brief Practice Fidelity Checklist
CATS-2: Child and Adolescent Trauma Screen Second Version
EBP: evidence-based practice
EBPAS-36: Evidence-Based Practice Attitude Scale
MCAR: Missing Completely at Random
ProQoL: The Professional Quality of Life scale
REML: restricted maximum likelihood

## Supplementary material

The supplementary material for this article can be found at http://doi.org/10.1192/j.eurpsy.2025.10032.
