# Supplementary material for: Evaluation of the feasibility and effectiveness of trauma-focused cognitive behavioural therapy for children and youth in Ukraine during the war
Source: Eur Psychiatry. 2025 Jul 1;68(1):e96. doi: 10.1192/j.eurpsy.2025.10032 (PMC12303773; doi:10.1192/j.eurpsy.2025.10032)
Supplement: Pfeiffer et al. supplementary material [file S0924933825100321sup001.docx]

**Online Supplement**

**Table S1**

*Prevalence rates of reported traumatic events, self- and caregiver report*

| Traumatic Event | Self-report F0  (*n* = 293) | Caregiver-report F0  (*n* = 265) |
| --- | --- | --- |
|  | *n* (%) | *n* (%) |
| War | 202 (68.94) | 188 (70.94) |
| Bullying | 139 (47.44) | 114 (43.02) |
| Other not specified event | 138 (47.10) | 125 (47.17) |
| Witnessing family violence | 122 (41.64) | 103 (38.87) |
| Accident/ injury | 105 (35.84) | 75 (28.30) |
| Experiencing family violence | 101 (34.47) | 70 (26.42) |
| Experiencing Community Violence | 99 (33.79) | 70 (26.42) |
| Witnessing Community Violence | 98 (33.45) | 55 (20.76) |
| Scary medical procedure | 86 (29.35) | 73 (27.55) |
| Sudden loss | 78 (26.62) | 65 (24.53) |
| Sexual violence/ abuse | 60 (20.48) | 33 (12.45) |
| Online bullying | 37 (12.63) | 17 (6.42) |
| Sudden loss | 78 (26.62) | 65 (24.53) |
| Natural disaster | 35 (11.95 | 33 (12.45) |
| Attacked with weapon | 34 (11.60) | 23 (8.68) |
| Online sexual violence/ abuse | 25 (8.53) | 8 (3.02) |

*Note.* 7+ age

**Table S2**

*Prevalence rates of reported traumatic events in preschool children (aged 3-6), caregiver-report*

| Traumatic Event | preschool sample F0  (*n* = 31) |
| --- | --- |
|  | *n* (%) |
| War | 22 (70.97) |
| Other not specified event | 20 (64.52) |
| Witnessing violent attack | 15 (48.39) |
| Witnessing family violence | 13 (41.94) |
| Sudden loss | 10 (32.25) |
| Experiencing family violence | 9 (29.03) |
| Witnessing Community Violence | 9 (29.03) |
| Scary medical procedure | 9 (29.03) |
| Natural disaster | 7 (22.58) |
| Experiencing Community Violence | 6 (19.35) |
| Accident/ injury | 5 (16.13) |
| Experiencing robbery | 4 (12.90) |
| Being touched on private parts | 3 (9.68) |
| Sexual violence/ abuse | 3 (9.68) |
| Violent attack | 3 (9.68) |
